# GSScore: a novel Graphormer-based shell-like scoring method for protein–ligand docking

**DOI:** 10.1093/bib/bbae201

**Published:** 2024-05-05

**Authors:** Linyuan Guo, Jianxin Wang

**Affiliations:** School of Computer Science and Engineering, Central South University, Rd. Lu Shan Nan, 410083, Changsha, P.R. China; Hunan Provincial Key Lab on Bioinformatics, Central South University, Rd. Lu Shan Nan, 410083, Changsha, P.R. China; School of Computer Science and Engineering, Central South University, Rd. Lu Shan Nan, 410083, Changsha, P.R. China; Hunan Provincial Key Lab on Bioinformatics, Central South University, Rd. Lu Shan Nan, 410083, Changsha, P.R. China

**Keywords:** Protein–ligand docking, Graphormer, Shell-like architecture, scoring method

## Abstract

Protein–ligand interactions (PLIs) are essential for cellular activities and drug discovery. But due to the complexity and high cost of experimental methods, there is a great demand for computational approaches to recognize PLI patterns, such as protein–ligand docking. In recent years, more and more models based on machine learning have been developed to directly predict the root mean square deviation (RMSD) of a ligand docking pose with reference to its native binding pose. However, new scoring methods are pressingly needed in methodology for more accurate RMSD prediction. We present a new deep learning-based scoring method for RMSD prediction of protein–ligand docking poses based on a Graphormer method and Shell-like graph architecture, named GSScore. To recognize near-native conformations from a set of poses, GSScore takes atoms as nodes and then establishes the docking interface of protein–ligand into multiple bipartite graphs within different shell ranges. Benefiting from the Graphormer and Shell-like graph architecture, GSScore can effectively capture the subtle differences between energetically favorable near-native conformations and unfavorable non-native poses without extra information. GSScore was extensively evaluated on diverse test sets including a subset of PDBBind version 2019, CASF2016 as well as DUD-E, and obtained significant improvements over existing methods in terms of RMSE, $R$ (Pearson correlation coefficient), Spearman correlation coefficient and Docking power.

## INTRODUCTION

Protein–ligand interactions (PLIs) play a crucial role in various biological activities and processes [1, 2]. In fact, over 70% of FDA-approved drugs from 2015 to 2020 rely on PLIs for their clinical efficacy [3, 4]. Understanding the complex structures formed by proteins and ligands is essential for unraveling cellular pathways. However, experimental methods for determining these complex structures are often technically challenging and expensive [5], leading to a growing interest in computational approaches, such as protein–ligand docking, to unravel patterns in PLIs. Virtual screening aims to predict the complex structure of protein–ligand interactions based on individual protein structures [6, 7]. The process typically involves two main steps: sampling and ranking [8]. In the sampling step, docking algorithms explore the potential binding modes of the ligand with respect to the protein, generating a set of poses. These poses represent different configurations of the ligand within the protein’s binding site. Subsequently, a scoring function is applied to evaluate and rank the sampled poses. A reliable scoring function assigns higher scores to poses that closely resemble the native conformation, enabling accurate prediction of the near-native complex structure.

With the increasing number of PLIs being discovered in biological functions, protein–ligand docking has made significant progress in various aspects. This includes the construction of docking benchmarks [9–11], the design of sampling algorithms [12, 13] and the development of docking programs such as Autodock Vina [14], DOCK [15], GOLD [16], MOE [17], Glide (in Schrodinger) [18], UCSF Dock [19], Surflex (in Sybyl) [20] and MDock [21]. However, accurately identifying near-native conformations from a large set of sampled decoys using an appropriate scoring function remains a long-standing challenge. Previous studies have classified scoring functions into four categories: force field-based, empirical, knowledge-based and machine learning-based [22, 23]. Force field-based scoring functions calculate the weighted sum of several physics-related energy terms, including intermolecular electrostatic energy, van der Waals energy and desolvation potential [19]. Empirical scoring functions each stand on an energy factorization model and estimate the binding affinity by a weighted sum of different terms in the model [22]. Knowledge-based scoring functions convert distance-dependent pairwise contact distributions into potentials using an inverse Boltzmann relationship [21]. Some hybrid scoring functions combine force field-based energy terms with knowledge-based energy terms, such as KECSA [24] and SMoG2016 [25]. With the rapid advancement of machine learning algorithms and their applications in bioinformatics, many machine learning-based scoring functions have been developed [26–33]. These machine learning-based scoring functions have shown remarkable improvements in the accuracy of binding free energy prediction on experimentally determined structures. Unlike traditional scoring functions that consider simple relationships between interface conformations and interacting energies, machine learning-based scoring functions can uncover complex nonlinear combinations of features for protein–ligand interfaces. This makes them more powerful than traditional scoring functions. However, many machine learning-based scoring functions still struggle to capture the details or high-order interaction patterns of the spatial arrangement at interfaces. To improve their accuracy, researchers have proposed various methods, such as incorporating additional features, using more sophisticated machine learning algorithms and exploring different types of data. Furthermore, combining different machine learning-based and traditional scoring functions has been suggested to further enhance the accuracy of binding free energy prediction [34].

Graph neural network (GNN) has been effectively applied to process graph data [35, 36], such as modeling molecular structures [37]. GNN is learned based on the structural features of a graph and the relationships between nodes. They iteratively aggregate information from nodes and their neighboring nodes to update the representation of each node. GNN models have a mechanism similar to propagating information, where each node updates its representation at each iteration step, taking into account the information from its neighboring nodes. This iterative process helps the model capture the complex relationships between nodes and the global contextual information in the graph. A typical model is the Graph Transformer [38]. The Graph Transformer is a graph neural network model that incorporates the concept of attention mechanism. It combines the ideas of GNN and Transformer to handle graph data. The Graph Transformer utilizes self-attention mechanism to learn dependencies and contextual information between nodes. Unlike traditional graph neural network models, it does not rely on fixed neighbor sampling or graph convolution operations. Instead, it calculates the relevance between each node and other nodes using self-attention mechanism and updates the representation of nodes based on their relevance. Graphormer [39] is an enhanced graph feature modeling method that builds upon the Graph Transformer for graph representation learning. It incorporates additional internal structural information of the graph into the self-attention module to incorporate more topological information. Specifically, Graphormer incorporates centrality measures of nodes, spatial properties of nodes and edge feature vectors into the self-attention module. By integrating these additional features, Graphormer aims to capture and leverage more diverse and informative aspects of the graph’s structure. This allows the model to better understand the relationships between nodes and their surrounding topology, leading to more effective graph representation learning. In addition, the shell-like or radial protein environment representation has been proven to be a superior feature for protein function prediction [40]. This approach involves partitioning the protein’s environment into multiple concentric circles or shells, with the ligand atom at the center. Each shell is then used to model the ligand atom’s interactions with its surroundings at different Euclidean distances. Therefore, different concentric circles represent different molecular environments at varying Euclidean distances. This environment representation is more effective in distinguishing different Euclidean distances compared with traditional graph-based modeling approaches, resulting in a more robust model with enhanced discriminative power.

In this paper, we propose a model called GSScore, which combines Graphormer and the shell-like architecture. The aim of GSScore is to identify near-native conformations of protein–ligand complexes from a large number of docking poses. GSScore partitions the protein environment into multiple shell regions centered around the ligand. Each shell region forms a separate graph, where the protein atoms within the shell interact with the ligand atoms. Multiple graphs are then processed using multiple Graphormer to learn graph representations and extract features. These features are fed into a Multi-Layer Perceptron (MLP) to predict the Root Mean Square Deviation (RMSD) value of the current pose. We evaluate the performance of the GSScore model on multiple datasets, including the PDBBind refined set [41], CASF2016 [11] and DUD-E [42]. The model’s overall performance is assessed using metrics such as RMSE, $R$-value, Spearman and Docking Power. These metrics provide insights into the accuracy, predictive power of the model. By the way, GSScore pays more attention to how a single small molecule binds to the target protein. Docking power. GSScore is more suitable for fine structural analysis of a single small molecule to the target protein, rather than the rough screening of a large number of various small molecules, which is the limitation of our model discussed in the Supplyment Materials.

## MATERIALS AND METHODS

### Datasets

The main dataset utilized in this study was the PDBbind database version 2019 [41] (PDBBind2019), comprising 17 679 protein–ligand complexes along with their corresponding RMSD values. It is worth noting that the RMSD values were calculated with spyrmsd program [43].

To ensure consistency with the train and test allocation strategy employed by DeepBSP [44], we employed the protein–ligand complexes from CASF2016 as the test dataset, consisting of a total of 285 complexes. Since these 285 complexes are already present in the PDBbind2019 data, and the poses in PDBbind2019 and CASF2016 are generated using different docking programs, we separated the complexes in PDBbind2019 with the same names as those in CASF2016 to form the Primary Test Set. The remaining complexes were assigned to the training set. In other words, the overlap complexes between training and testing were removed from training set. Additionally, CASF2016 was used as the Secondary Test Set in its entirety. As a result, a total of 177 300 poses from 11 925 complexes were employed for training. Furthermore, the training set (PDBBind2019) was evenly divided into four parts, and each part was trained separately, resulting in the creation of four models.

In order to ensure that each complex has at least one native ligand in every partition of the training set, we replicated the native ligand for each complex four times. In PDBbind2019, complexes that were flagged as redundant, lacking ligand fragments, missing protein pockets, involving covalent bonds between ligands and proteins, containing ligands with special elements and isomers were excluded from both the training and testing data. We also filtered out complexes with a resolution greater than 3.0 Å or NMR complexes to ensure the reliability of the initial structure. Additionally, complexes with too few heavy atoms and excessive rotatable bonds were excluded, and only complexes with 5–60 ligand atoms were retained, as these complexes are more suitable for the calculations performed by the docking software.

Although this study primarily focused on small molecules labeled as ligands in the PDBbind database, some complexes contain additional small molecules surrounding these ligands. These small molecules can influence the binding conformation of the ligand and should be considered part of the receptor. However, the receptor files provided in the PDBbind database do not include these small molecules. Therefore, we required the HETATM atoms present in the protein PDB file. Since the PDBbind data do not provide these atoms, we instead utilized the RCSB PDB files [45]. Consequently, instead of directly using the receptor and ligand files provided by the PDBbind database, we implemented the following approach with the complex PDB files downloaded directly from the PDB database. The original PDB files provided the accurate complex assembly states. In cases where there was no corresponding SDF file in the PDB database or the files were clearly erroneous (e.g. the number of atoms or coordinates inconsistent with those in the PDB file), we used the ligand Mol2 files provided by the PDBbind database as a substitute.

Once the complexes in PDBbind2019 were prepared, AutoDock Vina [14] was utilized to generate docking poses for each ligand in the complexes. In this process, the receptor PDB files and ligand Mol2 files were converted to PDBQT files using the MGTools program of AutoDockTools [46]. Before docking, all water molecules in the complexes were removed.

Additionaly, we utilized a third test dataset called DUD-E [42]. This dataset comprises 102 protein–ligand complexes with their crystal structures, along with additional ligand molecules that do not bind to the respective protein. DUD-E is specifically designed for evaluating molecular docking scoring functions. For DUD-E, we separated the protein and ligand molecules from the crystal structures and performed re-docking using AutoDock Vina [14], generating 100 poses for each complex. These poses were then assessed using the spyrmsd program to calculate their RMSD values, which were used for comparative testing.

Furthermore, we utilized the CASF2016 decoys of screening as the fourth test dataset for the screening power evaluation. According to the details of definition of screening power, these test data are based on cross-docking where one protein target corresponds to multiple different ligands. Therefore, we used the name CASF2016-CrossDocking to distinguish from the secondary test dataset. More details refer to supplementary materials part 3.

### RMSD distribution

To analyze and compare the experimental results in more detail, we separately calculated the RMSD distributions for the training set, primary Test, CASF2016 and DUD-E datasets, as shown in Supplementary Figure S1a–d, respectively. More details are in the supplementary materials part 1.

To quantitatively compare the differences between the primary test, CASF2016, DUD-E and the training data, we used Jensen–Shannon divergence [47]. The Jensen–Shannon divergence ranges from 0 to 1, where 0 indicates identical distributions and 1 indicates completely different distributions. We calculated the divergence for the range of RMSD less than 2.0Å (near-native) or RMSD less than 10.0Å, respectively, and the results are shown in Table 1. From the table, it can be observed that within the range of RMSD less than 2.0Å, the primary test and DUD-E datasets are significantly closer to the training data compared with CASF2016, with the primary test showing the highest degree of agreement with the training data within this range. Within the range of RMSD less than 10.0Å, the primary test still exhibits a higher level of proximity compared with CASF2016 and DUD-E. The analysis of the Jensen–Shannon divergence indicates that CASF2016 and DUD-E better reflect the model’s generalization capability.

**Table 1:** The Jensen-Shannon divergence between training and several test sets

	RMSD<2Å	RMSD<10Å
primary test	0.031	0.101
CASF2016	0.228	0.136
DUD-E	0.102	0.132

### Node and edge features

The node features used in this paper are presented in Table 2. The molecular features we used are referenced from LigPose [48], but we made slight adjustments by removing the covalent feature. All the features in Table 2 are processed using RDKit. In the graph construction process, the atoms of the protein or ligand are considered nodes. For protein atoms, there are seven categories, while for ligand atoms, there are eight categories. Each category is encoded using a one-hot encoding scheme. For example, there are seven categories for protein atoms based on their Atom types, with an additional ’other’ category. Hence, a one-hot vector of length eight is used to represent the category of a protein atom.

**Table 2:** The Node and edge features

protein node features	ligand node features	edge features
atom type(7)	atom type(7)	bond type(7)
atom degree(10)	atom degree(10)	distance(1)
implicit valence(6)	implicit valence(6)	
neighboring hydrogen(6)	neighboring hydrogen(6)	
hybridization(9)	hybridization(9)	
amino acid type(22)	formal charge(22)	
atom name(37)	ring size(12)	
	aromatic(2)	

The categorization of protein atoms and ligand atoms is different. Protein atoms are classified into H, C, N, O, P, S, metal (Ca, Fe, K, Mg, Mn, Na, Zn) and other categories. Ligand atoms are classified into H, C, N, O, P, S, halogen (F, Cl, Br, I) and other categories. In the protein features, the Atom type represents the element category of the atom, while the atom name represents the string representation of the atom in columns 13–16 of the PDB file.

The features Atom degree, Implicit valence, Neighboring hydrogen and Hybridization for ligand atoms are similar to those of protein atoms. The feature ’formal charge(22)’ for ligand atoms represents the formal charge of the atom. The feature ’ring size(12)’ indicates the size of the ring that the atom belongs to. If the atom is part of a fused benzene ring, the largest ring is selected. Currently, support is provided for rings with a maximum of 10 atoms, and anything beyond that is categorized as ’other’. The feature ’aromatic(2)’ represents whether the atom is aromatic or not.

In addition, we have defined seven categories for the interactions between atoms (edges between nodes): Single, Double, Triple, Aromatic, Non-covalent, Other and Unknown. These categories represent different types of interactions or bonds between atoms in the molecular structure. The Single, Double and Triple categories indicate single, double and triple covalent bonds, respectively. The Aromatic category represents aromatic interactions, often found in conjugated systems. The Non-covalent category encompasses non-covalent interactions such as hydrogen bonds, van der Waals interactions and electrostatic interactions. The Other category is used for any other types of interactions not covered by the previous categories. The Unknown category is assigned when the nature of the interaction is not known or cannot be determined. These interaction categories provide important information about the connectivity and bonding patterns between atoms in the molecular system.

### Shell-like graph

**Figure 1:**
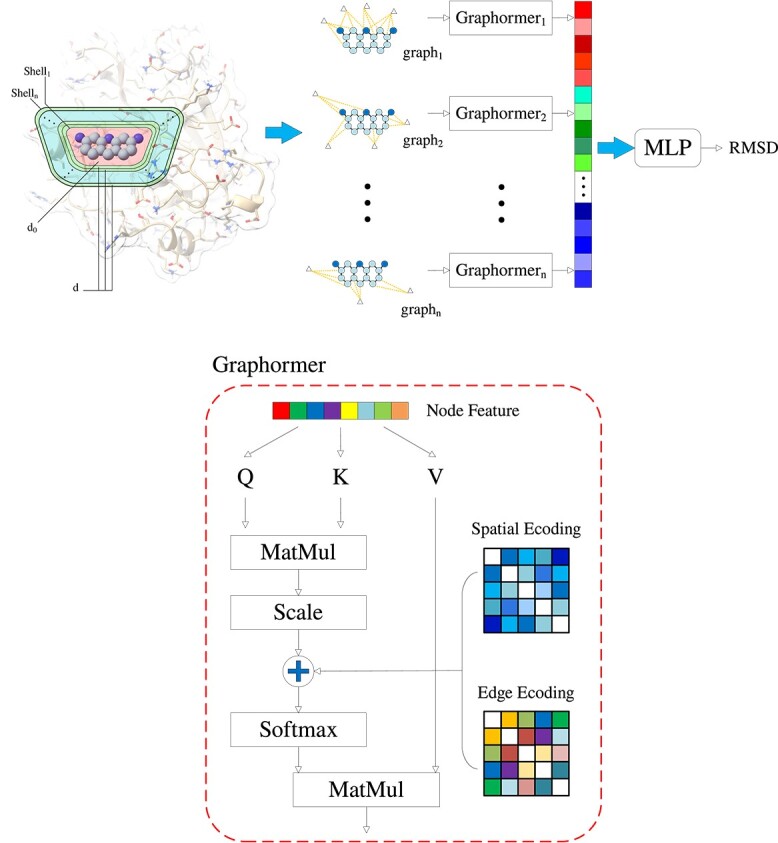
**The network architecture of GSScore**. For a given protein–ligand conformation, GSScore divides it into multiple layers of shells with different distances, using the ligand molecule as the central reference point. Each shell includes protein atoms that are in proximity to the ligand, and a separate graph is constructed for each shell. These graphs are then individually inputted into a Graphormer to extract their respective feature vectors. The resulting feature vectors from all the graphs are concatenated into a single long vector, which is then passed through an MLP layer to predict the RMSD. Graphormer is an enhancement of the traditional Transformer model, incorporating spatial topological features and edge features based on the shortest path. This integration of graph topology and edge features helps to improve the predictive power of the model.

The shell-like partitioning of the molecular environment in GSScore is inspired by the work of Wen Torng *et al.* [40] and DeepRMSD [34]. However, unlike DeepRMSD, we do not consider each atom as the center of a spherical shell. Instead, we use the entire ligand molecule as the center. Therefore, a shell in GSScore is not necessarily a spherical shell but can have irregular shapes. As shown in Figure 1, the entire ligand molecule in the protein pocket serves as the center of the shell, and multiple layers of shells are defined. The distance between the first shell and all ligand atoms is denoted as $d_{0}$, and subsequent shells are separated by a distance $d$ from the previous shell. There are a total of $n$ shells, and the distance between the $k$th shell and all ligand atoms is given by $d_{0} + (k-1)\times d$.

When processing the $k$th shell, a subgraph, denoted as graph$_{k}$, is constructed by considering the ligand atoms and all protein atoms between the $k$th and $(k-1)$th shells. In graph$_{k}$, the distance between ligand atoms is fixed to 1, and there are associated edges between protein atoms and ligand atoms within the distance range of DistMin to DistMax. When $k=1$, DistMin is set to 0, and DistMax is set to $d_{0}$. When $k>1$, DistMin is set to $d_{0}+(k-2) \times d$, and DistMax is set to $d_{0}+(k-1) \times d$. No edges are created between protein atoms to better capture the dependencies between protein and ligand atoms. The node and edge feature vectors for the subgraph graph$_{k}$ are described in section 2.3. Each node in the subgraph is represented by a vector of length 171. To differentiate between protein and ligand atoms, only the first 97 dimensions are used for protein atoms, with the remaining 74 dimensions padded with zeros. For ligand atoms, only the last 74 dimensions are used, with the first 97 dimensions padded with zeros. The edge feature vector is different from the node feature vector and has a total of eight dimensions. The first seven dimensions are a one-hot vector representing the edge type, and the last dimension represents the distance of the edge. Instead of using the actual Euclidean distance, the distance value is defined as DistMax.

### Network architecture of GSScore

GSScore inputs graph$_{k}$ into the corresponding Graphormer$_{k}$. The model architecture of GSScore is based on Graphormer [39]. The fundamental idea behind Graphormer is to use the topological information of the graph instead of the positional encoding used in the original Transformer. The basic equation of the Transformer is shown in Equation (1) 


(1) \begin{align*}& \begin{split} Q&=H W_{Q}, \quad K=H W_{K}, \quad V=H W_{V}, \\ A&=\frac{Q K^{\top}}{\sqrt{d_{K}}}, \quad \operatorname{Attn}(H)=\operatorname{softmax}(A) V, \end{split}\end{align*}

where $H=\left [h_{1}^{\top }, \cdots , h_{n}^{\top }\right ]^{\top } \in \mathbb{R}^{n \times d}$, $W_{Q} \in \mathbb{R}^{d \times d}$, $W_{K} \in \mathbb{R}^{d \times d}$, $W_{V} \in \mathbb{R}^{d \times d}$, $h_{i}^{\top } \in \mathbb{R}^{1 \times d}$ is the hidden representation at position $i$ and $d$ is the hidden dimension.

Here, we only used two additional graph topological features from the original Graphormer paper, namely Spatial encoding and Edge encoding, but we omitted the Centrality encoding feature [39]. In our specific experiments, we found that the Centrality encoding feature did not improve the performance of the model. A more detailed analysis of this will be presented in the ablation experiments.

For Spatial encoding, we use the shortest path between nodes $v_{i}$ and $v_{j}$ as the original feature, as shown in Equation (2). The function $\phi (v_{i}, v_{j}): V \times V \to \mathbb{R}$ is defined to represent the shortest path between nodes $v_{i}$ and $v_{j}$. We use the Floyd–Warshall algorithm [49] to compute the shortest paths between any two nodes. If there is no shortest path between the two nodes, we set the distance value to $-1$. Additionally, we set a maximum length for the shortest path, hop_max. If the shortest path between $v_{i}$ and $v_{j}$ is greater than hop_max, the path is truncated to the length of hop_max. Considering that the shortest path values have a large number of repeated values, directly incorporating them into the attention matrix is not a wise choice. Therefore, we introduce a learnable scalar $b$ as the bias for self-attention, where $\phi (v_{i}, v_{j})$ is mapped to a learnable parameter. With the inclusion of $b_{\phi (vi,vj)} \in \mathbb{R}$, each individual Transformer layer can adaptively adjust its receptive field based on the graph structure. This is different from traditional GNNs, which can only aggregate information from neighboring nodes. When $b_{\phi (vi,vj)}$ decreases during the training process, the model will pay more attention to nearby neighbor information, or otherwise, it will focus more on distant neighbor information.

For Edge encoding, we encode each edge along the shortest path between nodes $v_{i}$ and $v_{j}$, as shown in the right half of Equation (2). Traditional approaches for incorporating edge features either directly add the edge information to the node feature vectors or apply the edge information during the aggregation of node information. Both of these methods propagate the edge information to neighboring nodes but may overlook the contribution of edge information to the entire graph. Given two nodes $v_{i}$ and $v_{j}$ on the graph, we use the Floyd–Warshall algorithm to find the edge sequence $(e_{1}, e_{2},..., e_{N})$ along the shortest path between them. Each edge $e_{i}$ corresponds to a learnable parameter $w_{n}^{E}$, and the feature vector of each edge is computed by taking the dot-product with the parameter vector. After computing the dot-products for all edges along the path, the resulting value $c_{ij} \in \mathbb{R}$ is added to the attention matrix as the attention weight between nodes $v_{i}$ and $v_{j}$, along with $b_{\phi (vi,vj)}$. Similar to Spatial encoding, paths longer than hop_max are truncated to a length of hop_max 


(2) \begin{align*}& \begin{split} A_{i j}&=\frac{\left(h_{i} W_{Q}\right)\left(h_{j} W_{K}\right)^{T}}{\sqrt{d}}+b_{\phi\left(v_{i}, v_{j}\right)}+c_{i j}, \\ c_{i j}&=\frac{1}{N} \sum_{i=1}^{N} x_{e_{i}}\left(w_{i}^{E}\right)^{T}, \end{split}\end{align*}

where $x_{e_{i}} \in \mathbb{R}^{d_{E}}$ is the feature of the $i$th edge, $w_{i}^{E} \in \mathbb{R}^{d_{E}}$ is the $i$th learnable parameters, $d_{E}$ is the dimension of edge feature and $N \le hop{\_}max$ is the length of the shortest path between $v_{i}$ and $v_{j}$.

In addition, considering that in some conformations, due to the docking algorithm, the ligand molecule may be far away from the protein molecule, for example, the shortest distance between the ligand molecule and the protein molecule is more than 20Å. Therefore, we define that when there are none of the protein atom nodes in the multi-shell graph constructed by the protein–ligand complex conformation, it is considered that the predicted RMSD value of this conformation is infinity.

### Training GSScore

During training, AdamW optimizer with an initial learning rate of 0.001 was used to minimize the mean square error (MSE) loss between predicted RMSD and truth RMSD of the pose as equation 3 below. During the training process, we use a variable learning rate, where the learning rate is halved every $STEP$ epochs. After conducting extensive experiments, we found that setting $STEP$ to 25 works well. Additionally, we employ early stopping, which stops the training process if the loss does not decrease for a consecutive number of $STEP$ epochs. This helps to avoid unnecessary training. The maximum number of training iterations is set to 150, as we have observed through extensive experiments that the checkpoint models converge before reaching 150 iterations. There will be 150 checkpoint models at most. The training data are evenly divided into four parts, with one part used for training and another randomly selected part used for validation. The validation dataset is used to select the best checkpoint model from multiple epochs and evaluate it on the test dataset to calculate various performance metrics for the part of training data we selected above. There will be four models for we have four parts of training data. The mean value and standard deviation of the four models will be reported as final results 


(3) \begin{align*}& MSE=\frac{1}{n} \sum_{i=1}^{n}(\hat{y}_{i}-y_{i})^{2}.\end{align*}

### Evaluation metrics

Six basic evaluation metrics, RMSE, $R$, Spearman, Docking power, Hit rate and Enrichment factor are used to compare the performance of GSScore with other methods. $R$ means the Pearson’s correlation coefficient between the computed RMSDs and the experimental binding RMSDs, while Spearman means the Spearman correlation coefficient between the computed RMSDs and the experimental binding RMSDs.

Docking power refers to the ability of a scoring function to identify the native ligand binding pose among computer-generated decoys [11]. Ideally, the native binding pose should be identified as the top-ranked one. Docking power is usually described as the percentage of complexes that have near-native poses within top-ranked poses, can be described using the following equation: 


(4) \begin{align*}& \text{Docking power} = \frac{m(k)}{M},\end{align*}

where $m(k)$ is the number of complexes that have at least one near-native poses among top $k$ ranked poses, and $M$ is the total number of complexes in the test set.

Hit rate measures the fraction of near-native poses among top-ranked poses relative to all near-native decoys among the entire poses set for a given protein–ligand complex. Since there is not only one protein–ligand complex in the test sets, we use the mean Hit rate to measure the performance. Therefore, the mean Hit rate can be described below 


(5) \begin{align*}& \text{Hit rate} = \frac{1}{M} \sum_{i=1}^{M} \frac{h(k)}{P},\end{align*}

where $h(k)$ is the number of near-native poses among top $k$ ranked poses, and $P$ is the total number of near-native poses among the entire set of poses for a given protein–ligand complexes.

Enrichment factor (EF) is regarded as the second quantitative indicator of screening power for docking poses [11]. We use ’EF’ as the shorthand for EF. Screening power refers to the ability of a scoring function to identify the true binders to a given target protein among a pool of random molecules. The EF can be described below. 


(6) \begin{align*}& \text{Enrichment factor} = \frac{1}{M} \sum_{i=1}^{M} \frac{h(k)}{P \times \alpha (k)},\end{align*}

where $\alpha (k)$ is the fraction of $k$ to the total number of docking poses in a complex.

In addition, we also consider Screening power as an evaluation indicator. This evaluation index is divided into two parts, namely, Success rate and EF. The specific definition of Screening power can be found in the literature. Different from the previous six basic evaluation indicators, Screening power is aimed at cross-docking. In the docking process described above, a protein target corresponds to only one ligand, and the docking software searches for multiple poses for that ligand. During screening power docking, a protein target corresponds to multiple different ligands, which are derived from ligand molecules in different protein complexes. Therefore, screening power is used for cross-docking evaluation. It is worth to note that there are differences between re-docking and cross-docking for the EF, and we use the ’EF(re-docking)’ and ’EF(cross-docking)’ to distinguish them. The results of EF(cross-docking) are in the Part3 of the Supplementary Materials.

## EXPERIMENTS AND RESULTS

### Scoring functions to be compared

We have extensively evaluated our GSScore on the primary test set, CASF2016 test set and DUD-E test set, and compared it with three RMSD prediction functions of protein–ligand docking poses, DeepBSP [44], DeepRMSD [34] and ViTScore [50]. The reason why we chose these functions was that all of them were trained on PDBBind2019 general set [34, 44, 50]. Therefore, we can make a fair competition. DeepBSP was the first RMSD prediction function based on 3D-CNN. Its network architecture was similar to KDEEP [28] which was widely used in binding affinity prediction of protein–ligand docking poses. Meanwhile, we compared our previous RMSD prediction function ViTScore [50], which was based on the 3D Vision Transformer. In addition, we also compared our method with DeepRMSD [34]. DeepRMSD was the first method that applied the shell-like modeling approach to RMSD prediction. However, we thought that DeepRMSD+vina should not be used here for two main reasons. During the computation process, DeepRMSD+vina fine-tuned the spatial structure of poses, which was different from the conventional definition of a scoring function. The spatial structure changes of poses should be part of the searching process, and an independent scoring function should not alter the spatial structure of poses. DeepRMSD+vina progressively optimized the spatial structure of each pose during the iteration process, leading to some non-native poses being transformed into near-native poses. This resulted in an increase in evaluation metrics such as Docking power, Hit rate and EF, as the numerator increased. This evaluation approach might be not suitable for traditional scoring functions. Therefore, for fairness, we compared our method with DeepRMSD alone.

### Evaluation of GSScore on the primary test set

As shown in Table 3, GSScore performs the best among the four methods in terms of RMSE metrics. Specifically, GSScore achieves an RMSE as low as 1.531, which is 4.25% lower than our previous work, ViTScore and 4.97% lower than DeepBSP. However, DeepRMSD gives an RMSE of 6.971, which greatly exceeds our expectations. In the experimental process, although we have excluded some poses that encountered errors during the execution of DeepRMSD, there are still some poses that yield exceptionally large RMSD values when being inputted into DeepRMSD, despite no errors occurring during their execution. Therefore, we continue to exclude poses with predicted RMSD values greater than 15Åto avoid potentially misleading RMSE calculations. It is worth noting that there is no RMSE evaluation in the Vina method here, because the affinity value of each conformation calculated by Vina is not the RMSD value of each pose, which is different from the prediction results of other methods.

**Table 3:** Comparison of GSScore with DeepBSP, ViTScore, Vina and DeepRMSD for the primary test set^1,2^

$\textbf{Indicators} \bigg\backslash \textbf{Methods} $	DeepBSP	DeepRMSD	ViTScore	Vina	GSScore
RMSE	1.611	6.971	1.599	∖	**1.531$\ {\pm }\ $0.012**
R	0.826	−0.011	0.828	−0.068	**0.835$\ {\pm }\ $0.006**
Spearman	0.821	0.273	0.843	0.042	**0.881$\ {\pm }\ $0.007**
Docking power	0.902	0.697	**0.996**	0.674	0.895$\ {\pm }\ $0.015
Hit rate	0.128	0.106	**0.161**	0.086	0.139$\ {\pm }\ $0.005
EF(re-docking)	12.412	10.282	**15.675**	8.160	13.550$\ {\pm }\ $0.223

$^{1}$ ’∖’ means that there is no corresponding evaluation index for this method $^{2}$ Bold font indicates the maximum value in a row.

In Table 3, $R$ represents the Pearson coefficient between the predicted RMSD values and the true RMSD values for all poses. However, for Vina, $R$ represents the Pearson correlation coefficient between the predicted conformational affinity and the conformational RMSD value. It can be observed that GSScore achieves an $R$-value of 0.835, which is 0.007 higher than our previous work,ViTScore and 0.009 higher than DeepBSP. Although the improvement is not significantly pronounced, it is still a noteworthy enhancement considering a large number of test poses, exceeding 22 000. Additionally, it is widely recognized that for any evaluation metric, the closer it is to its optimal value, the more limited the room for improvement becomes.

In Table 3, Spearman represents the Spearman coefficient between the predicted RMSD values and the true RMSD values for all poses. It is also worth to note, for Vina, Spearman represents the Spearman correlation coefficient between the predicted conformational affinity and the conformational RMSD value. It can be observed that GSScore achieves an Spearman-value of 0.881, which is 0.038 higher than our previous work, ViTScore and 0.06 higher than DeepBSP.

For the Docking power, Hit rate and EF(re-docking) shown in Table 3, GSScore does not perform optimally on the primary test. This could be attributed to the model sacrificing some performance in order to improve generalization on test data with inductive bias or similar distribution. This is in contrast to our previous work, ViTScore, which exhibits significant performance on test data with similar distribution, resulting in excellent results on the primary test. However, it is important to note that GSScore does not perform poorly either. It achieves a Docking power of 0.895, which is close to DeepBSP; a Hit rate of 0.139, surpassing DeepBSP’s 0.128; and an EF(re-docking) of 13.550, which also exceeds DeepBSP’s 12.412.

### Evaluation of GSScore on CASF2016 test set

From Table 4, GSScore still has the lowest RMSE, highest $R$ and Spearman among the five methods. GSScore achieves an RMSE as low as 1.586, as well as $R$ and Spearman as high as 0.831 and 0.846, respectively, which are similar to the results in Table 3 and reflect the generalization ability of GSScore to some extent. Although GSScore has a Docking power of only 0.862, lower than ViTScore’s 0.905, DeepBSP’s 0.898 and Vina’s 0.902, its Hit rate of 0.047 and EF(re-docking) of 3.789 are both higher than the other three methods. It is worth noting that Docking power is more applicable for evaluating the performance of a ligand molecule in inverse virtual screening across multiple protein molecules. However, for the docking process of a ligand molecule on a single protein molecule, we pay more attention to the evaluation of the Hit rate and the EF in this study.

**Table 4:** Comparison of GSScore with DeepBSP, ViTScore, Vina and DeepRMSD for the CASF2016 test set

$\textbf{Indicators} \bigg\backslash \textbf{Methods} $	DeepBSP	DeepRMSD	ViTScore	Vina	GSScore
RMSE	1.601	2.239	1.764	∖	**1.586$\ {\pm }\ $0.016**
R	0.821	0.637	0.789	0.604	**0.831$\ {\pm }\ $0.008**
Spearman	0.808	0.657	0.843	0.528	**0.846$\ {\pm }\ $0.013**
Docking power	0.898	0.580	**0.905**	0.902	0.862$\ {\pm }\ $0.024
Hit rate	0.046	0.029	0.041	0.043	**0.047$\ {\pm }\ $0.003**
EF(re-docking)	3.665	2.284	3.277	3.635	**3.789$\ {\pm }\ $0.271**

$^{1}$ ’∖’ means that there is no corresponding evaluation index for this method $^{2}$ Bold font indicates the maximum value in a row.

Furthermore, in our previous work, ViTScore does not perform as well as DeepBSP in terms of RMSE and $R$ on CASF2016. Our previous analysis revealed that ViTScore is sensitive to the distribution of RMSD values, which leads to suboptimal performance on CASF2016, where the RMSD distribution differs from the training set. However, after extensive analysis, we have designed GSScore, which exhibits relatively good generalization. Thanks to the shell-like design, GSScore is able to capture the interaction patterns between atoms at different distance ranges. Despite the difference in RMSD distribution between CASF2016 and the training and primary test sets, GSScore is still capable of capturing the interaction patterns between ligand atoms and protein atoms at different distances.

### Evaluation of GSScore on DUD-E test set

The results on DUD-E test dataset are shown in Table 5. GSScore outperforms all other methods in all six evaluation metrics. In terms of RMSE, GSScore is the only method with an RMSE below 2Å, reaching as low as 1.668. It also has the highest $R$ and Spearman values among all methods, reaching 0.817 and 0.825. In terms of Docking power, GSScore surpasses the other three methods, reaching 0.85. This is an 18.1% improvement over our previous work, ViTScore and a 37.1% improvement over DeepBSP. Moreover, GSScore achieves a Hit rate of 0.161, which is 42.5% higher than DeepRMSD’s 0.113. GSScore also achieves an EF(re-docking) of 16.316, which is 42.4% higher than DeepRMSD’s 11.455.

**Table 5:** Comparison of GSScore with DeepBSP, ViTScore and DeepRMSD for the DUD-E test set

$\textbf{Indicators} \bigg\backslash \textbf{Methods} $	DeepBSP	DeepRMSD	ViTScore	Vina	GSScore
RMSE	2.258	14.151	2.010	∖	**1.668 ${\pm }$ 0.011**
$R$	0.596	−0.105	0.703	0.166	**0.817 ${\pm }$ 0.008**
Spearman	0.559	0.345	0.718	0.267	**0.835 ${\pm }$ 0.015**
Docking power	0.620	0.681	0.720	0.636	**0.850 ${\pm }$ 0.020**
Hit rate	0.101	0.113	0.108	0.091	**0.161 ${\pm }$ 0.003**
EF(re-docking)	10.147	11.455	10.926	9.216	**16.316 ${\pm }$ 0.252**

$^{1}$ ’∖’ means that there is no corresponding evaluation index for this method $^{2}$Bold font indicates the maximum value in a row.

From the previous analysis of RMSD distributions in supplymentary material, it is known that DUD-E has the lowest proportion of native poses among all test datasets. Therefore, correctly predicting the RMSD of native poses is a challenging task for all methods and should not be overlooked. The comprehensive superiority of GSScore over other methods on this dataset reflects its generalization ability. This also demonstrates, on a broader range of data, that the shell-like design approach helps improve the generalization performance of the model.

It is worth noting that DeepRMSD has an abnormally high RMSE and a negative $R$-value. In fact, during the experiments, we discovered that DUD-E yielded many abnormal RMSD predictions during runtime, similar to the primary test set. Similarly, we excluded poses with predicted RMSD greater than 15Å, as these can also be considered processing errors. The results show that DeepRMSD tends to overestimate the RMSD predictions for DUD-E, so although it does not exhibit abnormalities in terms of Docking power, the RMSE, $R$ and Spearman values cannot be considered normal.

## DISCUSSION

### Interpretability analysis

**Figure 2:**
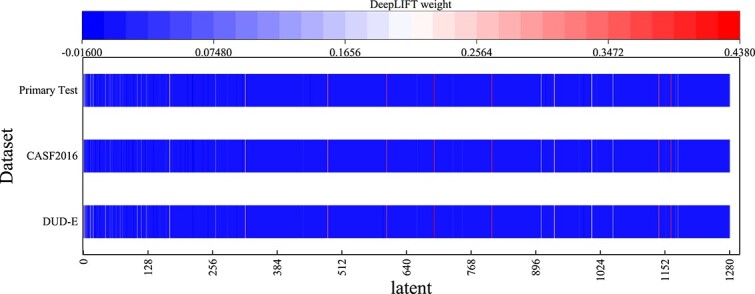
**Visualization of DeepLIFT weights**. The greater the weight, the more it tends to be red, and the reverse tends to be blue. The horizontal coordinate indicates the indexes of weights, and the vertical coordinate indicates different test data.

Here, we use the DeepLIFT [51] program to perform an in-depth analysis of the latent space of the model. Figure 2 shows the results of DeepLIFT analysis, where the $x$-axis represents the index of the embedding vector, and the $y$-axis represents different test datasets. We first input all the poses of a test dataset into GSScore. According to the model described in Figure 1, each pose passes through $n (n=10)$ Graphormer layers and obtains an embedding vector of length 128 for one Graphormer layer. This vector is then concatenated into one vector with a length of $128 \times n=1280$. DeepLIFT uses this 1280-length vector and the true RMSD value of the pose as inputs to analyze the weights of each dimension in the vector. Since multiple poses result in multiple weight outputs, we take the average of the weights for each dimension as the final analysis result.

We have intentionally divided the $x$-axis into 128 evenly spaced intervals to correspond to the 128-length embedding vector after $n (n=10)$ Graphormer layers. The larger the weight, the more it tends toward red, or otherwise, it tends toward blue. From the figure, it can be observed that the majority of the weights are very small, with only a few dimensions having larger weights. However, regardless of the test dataset, the distribution pattern of the weights is highly similar, with the larger weights appearing in the same dimension indices. It is worth noting that within each 128-length interval, there is at least one dimension with a high weight. This to some extent indicates that each Graphormer produces effective dimensions in the embedding vector, meaning that each shell’s subgraph can extract useful embedding features, thereby improving the predictive ability of the model. The positive weight representation of DeepLIFT has an increasing effect on the prediction results. In contrast, a negative weight indicates a reduced effect on the predictive results.

To visually demonstrate the embedding effect of GSScore’s latent space, we used t-SNE [52] to visualize over 10 000 data points. These data points are derived from the primary test, CASF2016 and DUD-E datasets. We combined the three datasets at the compound level and randomized the order. Then, we extracted multiple compounds until the total number of poses exceeded 10 000.

**Figure 3:**
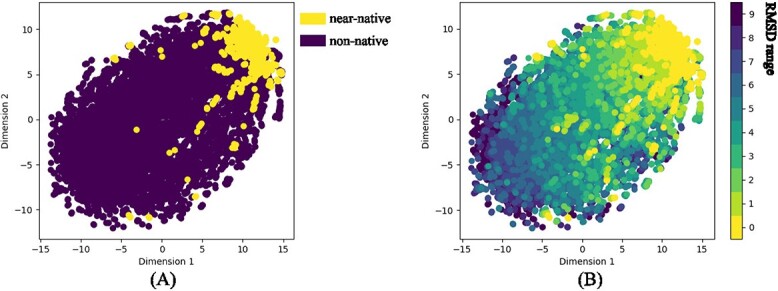
**Visualization of latent space with t-SNE**. **(A)** represents the case where only near-native poses and non-native poses are distinguished. Near-native poses are defined as poses with an RMSD less than 2A. **(B)** represents the case where poses are divided into 10 types. Each interval has a range of 1A, where poses with an RMSD of 0 represent native poses, and poses with an RMSD of 9 or above are grouped together in one category.

In Figure 3A, we distinguished between near-native poses and non-native poses. Near-native poses were defined as those with an RMSD less than 2Å. In Figure 3B, we divided the poses into nine intervals. Each interval had a 1Å interval, with native poses labeled as RMSD equal to 0, and poses with RMSD greater than or equal to nine grouped into a single category.

From the visualization in Figure 3A, it can be observed that the majority of near-native poses are concentrated in the upper right corner, while non-native poses are distributed in other regions apart from it. To further visualize the model’s performance, we created the visualization in Figure 3B. It can be seen that poses from different intervals transition smoothly from the upper right corner to the lower left corner, showing a gradual change in color depth. This to some extent demonstrates that GSScore is not only capable of distinguishing near-native from non-native poses but also able to identify RMSD values of poses in multiple fine-grained intervals, which aligns with the design of GSScore using MSE loss.

**Figure 4:**
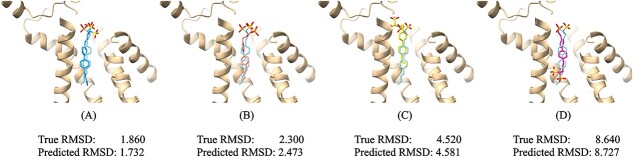
**Visualization of RMSD predictions**. The PDB ID: 2zcr. There are four different poses with different RMSD values. The native pose is represented by cyan in each subfigure, while other poses are represented by four different colors. The true RMSD and predicted RMSD values are under each subfigure.

Figure 4 is a visual example of the GSScore prediction for RMSD. The PDB ID of the protein–ligand complex is 2zcr. In the figure,A–D, respectively, correspond to four different poses. The true RMSD value and the predicted RMSD value for each pose are below it. The cyan pose in each image is the native pose, the gold ribbon in the background is the target protein, and the four different poses are represented by blue, orange, yellow and fuchsia, respectively. As can be seen from the figure, GSScore can have better prediction results for near-native pose of subfigure (A) or non-native poses of other subfigures.

### Ablation study

We conducted an ablation analysis on GSScore, comparing the effects of different features and network structures used in the model. Due to the relatively long training time, we limited the ablation experiments to the primary test set and tested only the model trained with a specific set of training data. The purpose of the ablation analysis was to gain a deeper understanding of the contributions of individual components and design choices in the GSScore model. By performing these experiments, we aimed to assess the significance of different features and network structures on the model’s performance and identify key factors that influence its effectiveness.

Table 6 presents the results of the ablation experiments on the Centrality, Edge and Spatial encoders in Graphormer. The first row indicates the absence of any Graphormer encoder, while the subsequent rows represent different combinations of encoders. From the table, it is evident that the Centrality encoding is generally not a beneficial feature and can even have a detrimental effect, which is why we did not include it in the model. When the Edge Encoding and Spatial Encoding are used individually, they contribute to some extent of performance improvement. However, their combined usage results in a more pronounced enhancement. Surprisingly, when the Centrality Encoding, Edge Encoding and Spatial Encoding are used together, the performance of the model deteriorates. It is worth noting that while RMSE and $R$ are typically positively correlated, this relationship may not hold true for Docking power, Hit rate and EF. For instance, comparing the first and last rows in Table 6, lower RMSE does not necessarily indicate higher Docking power. Similarly, contrasting the fourth and fifth rows, higher Docking power does not necessarily translate to higher Hit rate and EF.

**Table 6:** Ablation study for Graphormer Encoders

Centrality Encoding	Edge Encoding	Spatial Encoding	RMSE	$R$	Docking power	Hit rate	EF(re-docking)
			1.757	0.802	0.758	0.116	11.207
*			1.915	0.754	0.719	0.111	10.684
	*		1.654	0.809	0.810	0.130	12.703
		*	1.663	0.809	0.764	0.120	11.698
*	*		1.828	0.742	0.726	0.130	12.013
*		*	1.831	0.733	0.722	0.111	10.802
	*	*	**1.529**	**0.835**	**0.889**	**0.139**	**13.580**
*	*	*	1.762	0.775	0.778	0.112	10.855

$^{1}$ * means the corresponding Encoding features have been used. $^{2}$ Bold font indicates the maximum value in a column.

**Figure 5:**
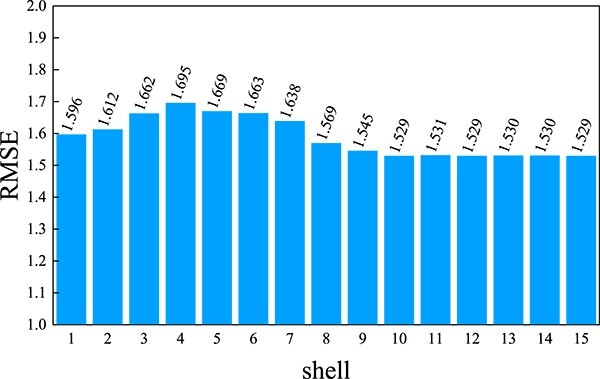
**RMSE results of primary test within different number of shells in GSScore**. The horizontal coordinate indicates the number of shells, and the vertical coordinate indicates the RMSE result of the primary test.

Additionally, we conduct an analysis of the impact of the number of shells on the model’s performance, as shown in Figure 5. The $x$-axis represents the number of shells in GSScore, which also corresponds to the number of Graphormers used. The $y$-axis represents the RMSE values on the primary test set. Due to the complexity of the experiments, we focused only on the influence of the number of shells on RMSE when using the Edge Encoding and Spatial Encoding in Graphormer.

From the figure, it can be observed that even with just one shell, which corresponds to the distance range of $d_{0}$ in Figure 1, GSScore already improves the model’s performance, surpassing the effectiveness of DeepBSP. However, as the number of shells increases, the performance of GSScore deteriorates until reaching a shell number of 8, where it starts to improve again. Beyond 10 shells, the model’s performance plateaus, and no further improvement is achieved. In the actual experimental process, when the number of shells exceeds 10, the batch size during training needs to be set below 32 to avoid GPU memory overflow issues. Considering both model performance and program efficiency, we selected a shell count of 10 as the optimal choice. The series of ablation experiments described above provide us with a deeper understanding of the impact of each module on the model’s performance. Based on these findings, we make informed decisions regarding the model structure, as depicted in Figure 1.

## CONCLUSION

We have developed GSScore, a deep learning model that combined Graphormer features and network architecture with a shell-like design approach for predicting the RMSD values of protein–ligand conformations. GSScore focuses on the environment of the ligand and divides it into multiple shells based on different distance ranges. Within each shell, protein atoms are used to construct a graph, resulting in multiple graphs that are input into different Graphormer modules. These modules generate multiple embedding vectors, which are then concatenated into a long vector and fed into an MLP layer for predicting the conformational RMSD value.

The features and network architecture of Graphormer enable GSScore to effectively capture the topological characteristics of atomic graphs in protein–ligand conformations. Furthermore, the shell-like design approach further enhances the generalization performance of GSScore. The results tested on the primary test, CASF2016 and DUD-E datasets, reveal that GSScore has better performance in predicting conformational RMSD values in terms of RMSE and $R$-value and GSScore does not achieve the best results in Docking power, Hit rate, EF and Screening power in all testing data sets.

Currently, GSScore utilizes only the raw chemical feature information as node and edge features and does not incorporate external information such as sequence conservation [53] and co-evolution [54]. Therefore, future work will consider integrating such information and differentiating between coarse-grained and fine-grained graph construction approaches to further enhance the prediction performance of protein–ligand conformational RMSD values.

**Key Points**

- We proposed a novel protein–ligand scoring function that ranked the docking poses based on the predicted RMSD value of each pose relative to the native ligand structure.
- The Graphormer model can effectively recognize the interaction patterns between protein atoms and ligand atoms in different distance ranges around ligand molecules.
- The shell-like architecture enabled multi-subgraph construction between ligand atoms and surrounding protein atoms in different distance ranges, allowing our model to handle interactions over long distances.

## Supplementary Material

Supplementary_Materials_bbae201

## Author Biographies

**Linyuan Guo** is a doctoral student at Central South University.

**Jianxin Wang** is a Professor at Central South University and the director in Hunan Provincial Key Lab on Bioinformatics.
