# Supplementary material for: GSScore: a novel Graphormer-based shell-like scoring method for protein–ligand docking
Source: Brief Bioinform. 2024 May 5;25(3):bbae201. doi: 10.1093/bib/bbae201 (PMC11070652; doi:10.1093/bib/bbae201)
Supplement: Supplementary_Materials_bbae201 [file supplementary_materials_bbae201.docx]

GSScore: a novel Graphormer-based Shell-like scoring method for protein-ligand docking

Supplementary materials

**Part 1. RMSD distribution of training and testing data sets.**


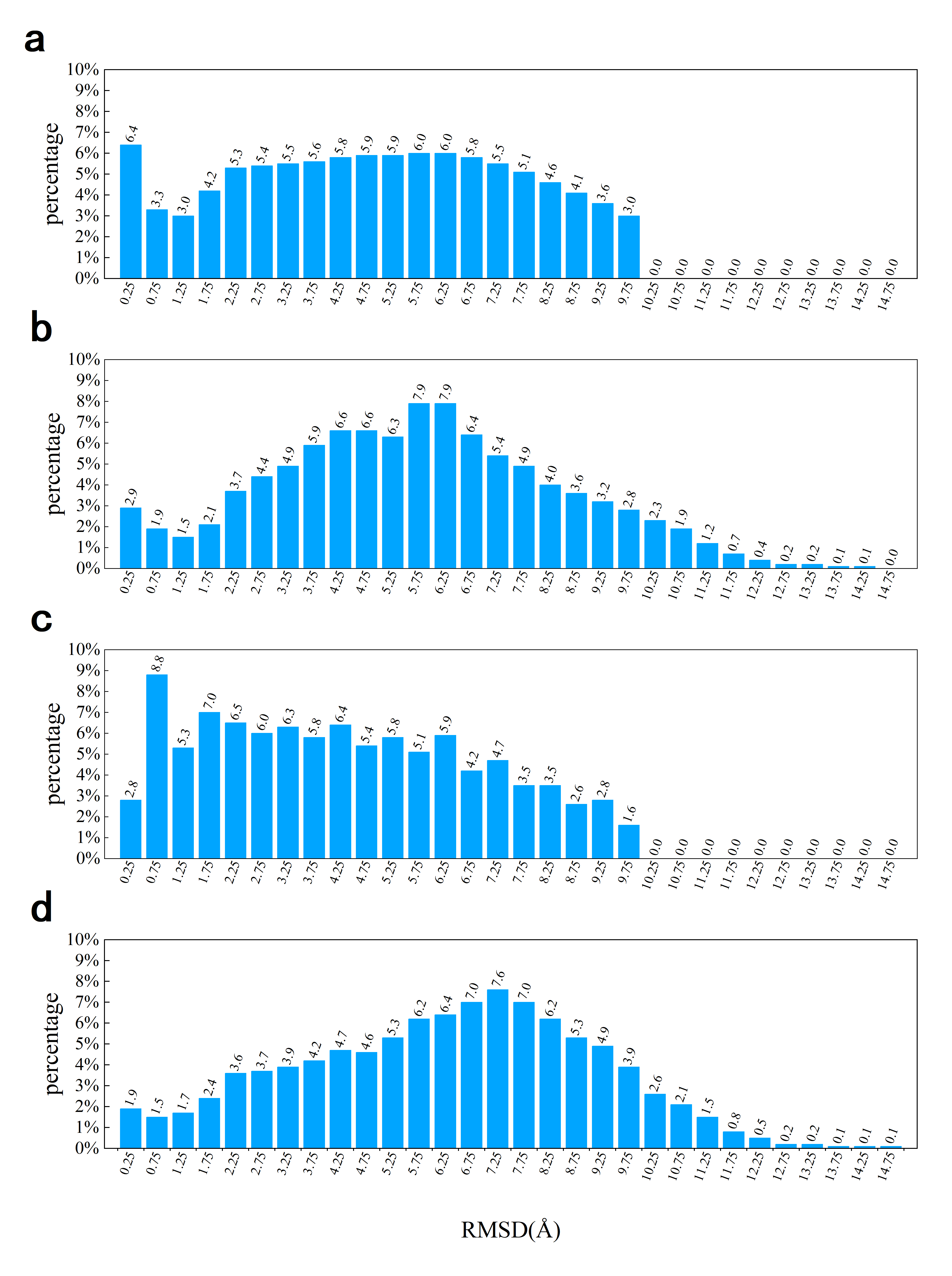


**Figure S1.** RMSD distribution of training, Primary, CASF2016 and DUD-E test sets. The number on the X-axis represents the center of the bins. The native poses are included in All of them above. (a) RMSD distribution of training poses; (b) RMSD distribution of Primary Test poses; (c) RMSD distribution of CASF2016 poses; (d) RMSD distribution of DUD-E poses.

To analyze and compare the experimental results in more detail, we separately calculated the RMSD distributions for the training set, primary Test, CASF2016, and DUD-E datasets, as shown in Figure S1(a), (b), (c), (d) respectively. For the complexes in the training set, some of them lacked any docking conformations with an RMSD less than 3.0Å, and these complexes were removed from the training set. Furthermore, all training set conformations with an RMSD greater than 10.0 Å were also removed. The RMSD distribution of remaining conformations is shown in Figure S1(a). The X-axis represents the range of RMSD values, and the Y-axis represents the percentage.

From this figure, we can observe significant differences in the RMSD distributions between the primary test and the CASF2016 test set. The RMSD distribution of the training set is relatively similar to that of the primary test, while CASF2016's distribution differs from the other two datasets. CASF2016 contains a relatively large number of decoys with RMSD values in the range of [0.5, 1.0). This aligns with the original intention of CASF2016, which aimed to "a new test set is compiled, which consists of 285 protein-ligand complexes with high-quality crystal structures and reliable binding constants."^[1]^. Clearly, within the range of [0.5, 1.0), the training set and primary test set have the fewest number of decoys. Therefore, the ability to fit CASF2016 based on the characteristics of the data distribution is crucial for evaluating the generalization ability of various RMSD prediction methods.

Additionally, since the testing data in DUD-E also comes from AutoDock Vina, its RMSD distribution is similar to that of the primary test. However, it is worth noting that both the primary test and DUD-E sets contain poses with RMSD greater than 10.0Å, while such poses are absent in the training set. Accurately predicting the RMSD for these poses demonstrates the generalization ability of each method. Furthermore, the percentage of poses within the range of [0, 0.5) in DUD-E is less than 2%, indicating a relatively small proportion of native poses in DUD-E, as the majority of poses within the [0, 0.5) range are native poses. Therefore, accurately predicting the RMSD of native poses in the DUD-E test set also serves as hard work for RMSD prediction methods.

**Part 2. GSScore architecture**

In order to explain the GSScore structure more simply and easily, we take a pose processing flow as an example here, and analyze GSScore in combination with Materials and Methods of the paper. As shown in Figure 1 in the main text.

1. When a protein-ligand conformation is given, GSScore establishes a three-dimensional rectangular coordinate system with the geometric center of ligand molecule as the origin.

2. Calculate the feature vector of each protein atom and ligand atom, which can be referred to Table 1 of the paper.

3. Using shell as a unit, divide protein atoms in different shells with ligand atom distance, so as to construct subgraph corresponding to different shells. For example, if it is now the i-th shell, enumerate all protein atoms and calculate their Euclidean distance from all ligand atoms. If the distance is within the i-th shell range, then the current protein atom forms an edge with the current ligand atom. The length of the edge is the thickness of the i-th shell, and the thickness of the shell can be calculated by the equation in the paper. The property of the side is non-covalent. It is worth noting that there is no edge between any two protein atoms.

4. The subgraphs constructed under different shells with protein-ligand conformation are put into their respective Graphormer models, so as to extract the embedding vector corresponding to each subgraph. These embeddings are concatenated as the final embeddings of the current input protein-ligand conformation.

5. Finally, the concatenated embedding vector is input into an MLP layer to predict the RMSD value of the current conformation.

Here are some hyperparameters setting below:

**Table S1**. Hyperparameters setting

| d0 | 5 |
| --- | --- |
| D | 0.5 |
| Number of shell (k) | 10 |
| Hop_max | 8 |
| Number of Graphormer | 10 |
| Number of each Graphormer layer | 16 |
| Number of MLP | 1 |

As for Graphormer's detailed treatment process, the paper gives a detailed explanation and will not be repeated here. If further details are needed, please refer to Ref.^[2]^.

**Part 3. Evaluation of Screening power on CASF2016 cross-docking test set**

Here, we introduce three of the most advanced or popular conformation scoring functions for comparison with the screening power evaluation indicators at CASF2016. Table S2 shows the evaluation results of Screening power by RTMScore, DeepRMSD+Vina, Vina and GSScore. By definition, Screening power is divided into two indicators: Success rate and Enrichment factor. As can be seen from Table S2, RTMScore is the best scoring function in the current Screening power results, while GSScore is quite different from it. However, this is understandable. Because Screening power is based on cross-docking, one protein target corresponds to multiple different ligand molecules, and each ligand molecule produces multiple search conformations. This is different from the previous training and test data. The previous training and test data are based on re-docking where one protein target corresponds to only one ligand molecule. Therefore, from the point of view of machine learning, GSScore does not obtain cross-docking training data, so GSScore is not as good as the methods of state of the art in Screening power.

**Table S2.** Comparison of Screening power with RTMScore, DeepRMSD+Vina and Vina for the CASF2016 cross-docking set.

|  | Success rate | EF(cross-docking) |
| --- | --- | --- |
| RTMScore | 0.667 | 28 |
| DeepRMSD+Vina | 0.474 | 21.95 |
| Vina | 0.298 | 7.7 |
| GSScore | 0.211 | 4.35 |

Based on this question, we conducted an in-depth analysis. We downloaded 57 targets and related ligand crystal structures from the PDB database^[3]^; then we took out the decoys corresponding to these 57 targets from the cross-docking data in CASF2016, and calculated the RMSD with the crystal structure ligand molecules (the crystal structure downloaded from PDB was superimposed with the structure in CASF2016), and the distribution of RMSD is shown in Figure S2.


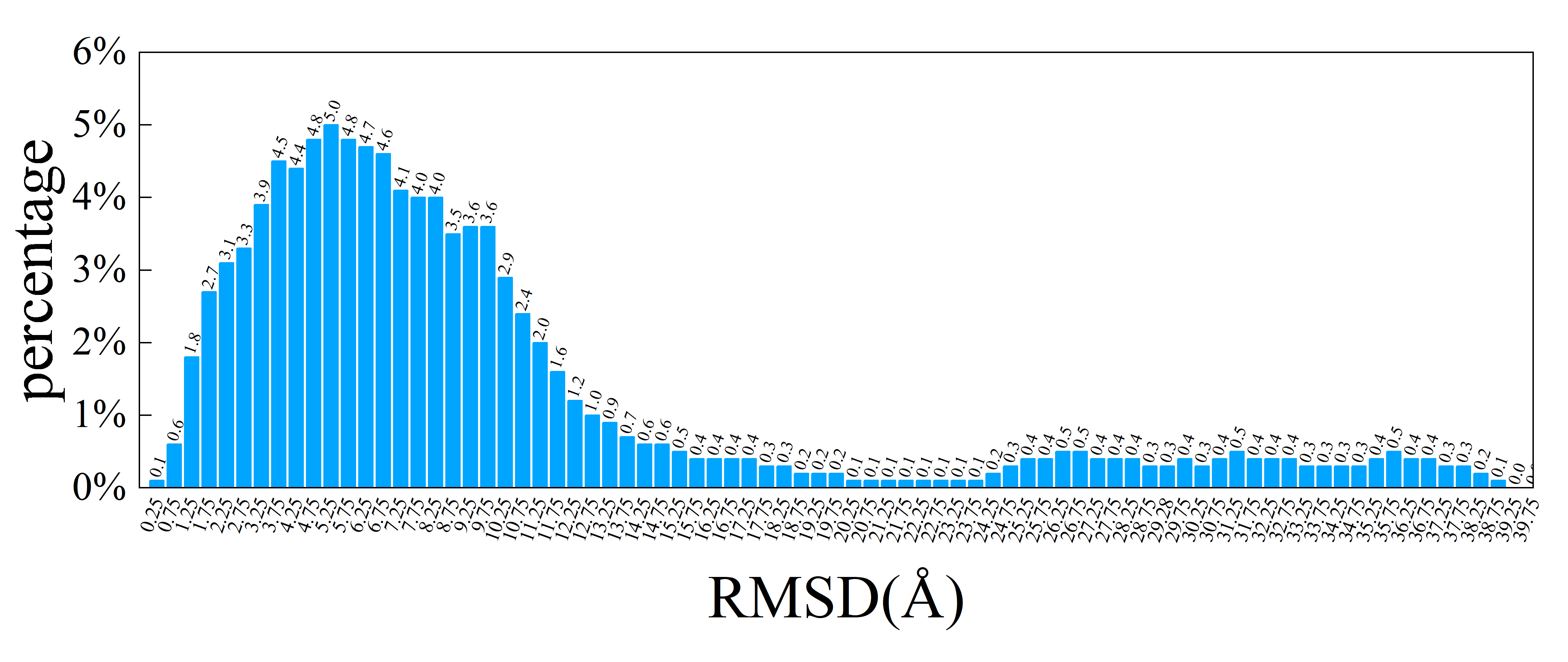


**Figure S2.** RMSD distribution of cross-docking decoys

As can be seen from Figure S2, the distribution of RMSD is quite different from the previous data. Notably, the RMSD of some poses exceeds 20 Å. We found through observation with PyMol or Chimera software that most of these poses did not dock in the pocket of the target protein, as shown in Figure S3 (A)(B)(C)(D). Obviously, these ligands did not really dock into the target protein. This leads to the generation of some abnormal graph data during the process of constructing the graph. These abnormal data will cause the model to output some extremely large or small RMSD prediction values, for example, the RMSD values predicted in Figure S3 (A)(B)(C)(D) are all 0.03. These abnormal values ultimately lead to poor ranking results in cross-docking, so the evaluation results of Screening power are also not good.


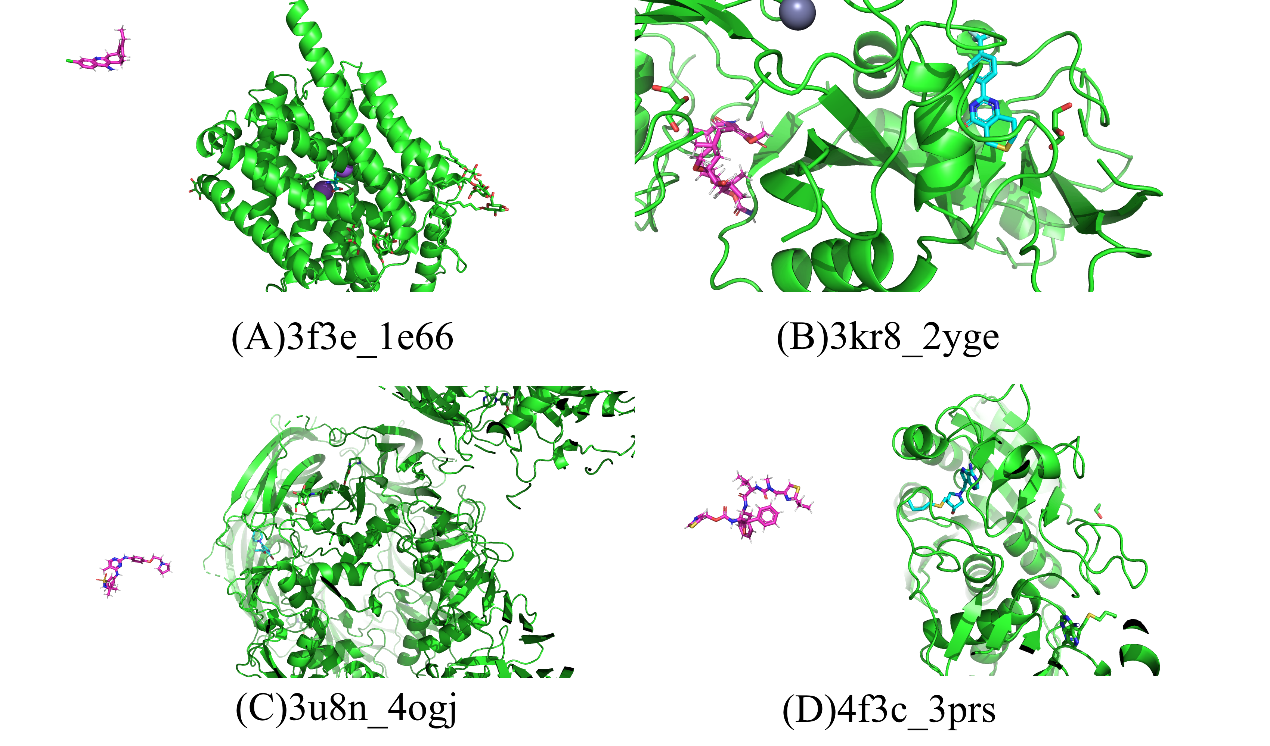


**Figure S3.** Illustrations of some abnormal protein-ligand docking poses in cross-docking decoys. The figure shows the PDB ID of the target protein and the cross-docking ligand molecule. Green represents the target proteins; cyan represents the native ligands of the crystal structures of the target proteins; amaranth represents the ligands from other proteins of cross-docking.

To further analyze the model, we conducted the following experiments. Considering that there are almost no cross-docking conformations in our training data from PDBBind2019, we tried to randomly extract about 30% of targets from the 57 targets of CASF2016 cross-docking as additional training data, and the remaining about 70% as Screening power test data. This experimental process was repeated 4 times, and the test results are shown in Table S3. The results in Table S3 show that when relevant cross-docking data is added, the results increase in the Screening power. Compared with Table S2, the average Success rate is increased from 0.211 to 0.365, and the average Enrichment factor is increased from 4.35 to 9.91. GSScore also has a certain conformation scoring ability in cross-docking data. The problem is that we need more training data about cross-docking.

**Table S3.** Comparison of Screening power with RTMScore, DeepRMSD+Vina and Vina for the CASF2016 cross-docking set with additional training data from CASF2016 cross-docking.

|  | Success rate | EF(cross-docking) |
| --- | --- | --- |
| RTMScore | 0.667 | 28 |
| DeepRMSD+Vina | 0.474 | 21.95 |
| GSScore | 0.365 | 9.91 |
| Vina | 0.298 | 7.7 |

In cross-docking, screening power is more concerned with which ligand in the current ligand database can form complex relationships with target proteins, but is not concerned with how these ligands bind to target proteins. GSScore is more concerned with which of the poses produced by a ligand docking on a target protein are closer to the binding pattern of the true crystal structure. In other words, GSScore is more concerned with how the ligand binds to the target protein. In the whole virtual screening process, we not only care about which small molecular could be an active molecular to the target protein from a large pool of molecules, but also care about how one molecular docking docks into the target protein. GSScore is more suitable for the latter.

**Part 4. More details about interpretability analysis of DeepLIFT.**

**Table S4.** Statistics of DeepLIFT weights.

| Property  Data set | >0 | =0 | <0 |
| --- | --- | --- | --- |
| Primary test set | 779 | 90 | 411 |
| CASF2016 | 736 | 110 | 434 |
| DUD-E | 771 | 95 | 414 |

As can be seen from Table S4, most of the weights calculated by DeepLIFT are greater than zero. It can be seen that no matter which test data, the distribution of weights is very similar. Although the number of these weights is not large, it cannot be ignored, which indicates that some features or network structures are redundant, and this is what we will consider in our future works.

**Part 5. Parameters of running Autodock Vina.**

When running Vina, we set the center to be the geometric center of ligand from crystal structure (native). Size_x,size_y, and size_z are all set to 20. Num_modes is set to 10, and exhaustiveness is set to 100. Since a single run of Vina only produces 10 different conformations, we run Vina 10 times with 10 different random seeds (seed) to produce 100 conformations.

**Reference**

[1] Minyi Su, Qifan Yang, Yu Du, et al. Comparative Assessment of Scoring Functions: The CASF-2016 Update [J]. Journal of Chemical Information and Modeling, 2019, 59(2): 895-913.

[2] Chengxuan Ying, Tianle Cai, Shengjie Luo, et al. Do transformers really perform badly for graph representation? [J]. Advances in Neural Information Processing Systems, 2021, 34: 28877-28888.

[3] Stephen K Burley, Helen M Berman, Charmi Bhikadiya, et al. RCSB Protein Data Bank: biological macromolecular structures enabling research and education in fundamental biology, biomedicine, biotechnology and energy [J]. Nucleic Acids Research, 2019, 47(D1): D464-D474.
